# Provision and continuation of antiretroviral therapy during acute conflict: the experience of MSF in Central African Republic and Yemen

**DOI:** 10.1186/s13031-018-0161-1

**Published:** 2018-07-02

**Authors:** Cecilia Ferreyra, Daniel O’Brien, Beatriz Alonso, Abdulbasset Al-Zomour, Nathan Ford

**Affiliations:** 1 Médecins sans Frontières Spain, Barcelona, Spain; 2 Médecins sans Frontières, Amsterdam, Netherlands; 3 National AIDS Program, Sanaa, Yemen; 4 Centre for Infectious Disease Epidemiology and Research, University of Cape Town, Cape town, South Africa

**Keywords:** HIV, Conflict, Contingency plan, Emergency response

## Abstract

**Background:**

Unstable settings present challenges for the effective provision of antiretroviral treatment (ART). In this paper, we summarize the experience and results of providing ART and implementing contingency plans during acute instability in the Central African Republic (CAR) and Yemen.

**Case presentation:**

In CAR, MSF has provided HIV care in three conflict-affected rural regions; these were put on hold throughout the acute phase of violence. “Run-away bags” containing 3 or 4 months of ART were distributed to patients at MSF facilities. Among 1820 HIV patients enrolled into care, 1440 (79%) initiated ART. By December 2016, 782 (54%) patients were still under ART, 354 (25%) have been lost to follow up and 182 (13%) had died. In 2013, when violence disrupted services, 683 patients were receiving ART. Between September–December 2013, 594 (87%) patients received runaway bags and by February 2014, 313 (53%) of these patients returned to the clinic.

In Yemen, when violence erupted, patients received a health card that included a helpline to call in case of drug shortages in admission to emergency stocks; this was not possible in CAR due to lack of a functioning telephone network. One thousand six hundred fifty-five PLWHA have been enrolled in care and 1470 (89%) initiated ART; 1056 (72%) are still followed on ART, 126 (9%) were lost to follow up, and 288 (20%) died. In January 2011 clashes began and by April 2011 MSF medical activities were interrupted. Of the 363 patients receiving ART, 363 (100%) received emergency bags to cover 9 months and by February 2012, 354 (98%) patients returned to care. In March 2015 a new wave of conflict affected Yemen, forcing HIV activities to revert to contingency planning.

**Conclusions:**

This experience provides further evidence that provision of HIV treatment and emergency drug stocks can be successfully provided to most patients in both conflict-affected settings.

## Background

Globally, an estimated 36.7 million people were living with HIV at the end of 2017 and 21 million of them were on antiretroviral therapy (ART). While this reflects substantial progress in scaling up access to care and treatment, still around half of the patients in need of ART have yet to be diagnosed or enrolled into care [1]. Among the many challenges faced in reaching more people with HIV, populations affected by humanitarian emergencies are particularly underserved. In 2014 it was estimated that 1.6 million people living with HIV were affected by humanitarian emergencies, of them 81% were in sub-Saharan Africa and almost two thirds (1 million) did not have access to ART [2].

Conflict-affected settings present a particular challenge for the effective provision of HIV care for a multitude of reasons, including the impact of the conflict on the local and national health systems, population movement, and the enduring view among providers of care that the provision of ART in these settings is not feasible [3–5]. Furthermore, concerns have been raised that providing ART could cause harm by putting patients at risk of drug resistance if ART was interrupted. Limited epidemiological data and programme experiences and lack of political will to prioritize the needs of displaced or conflict affected populations have all contributed to neglect HIV services in these settings [6]. Restricted movement can further deter clinic visits during periods of conflict, limiting access to care and treatment [7].

Data on the feasibility and impact of providing ART in conflict and emergency settings are limited. During the post-election conflict in Kenya during 2007–2008, treatment interruption was 71% higher compared to the period prior to the conflict, with Médecins sans Frontières (MSF) reporting around 30% of patients missing pills [8, 9]. A report from 22 MSF programs where ART was initiated in conflict or post-conflict settings, 64% of patients remained on ART with a rate of loss of follow up of 11% [10, 11].

MSF works in conflict-affected settings responding to the acute needs of these populations during the collapse of health services. In some of these settings HIV prevalence is high, leading to considerable HIV-related health needs. In many of these settings without access to HIV services, HIV-associated mortality is very high among hospitalized patients [12, 13].

In this paper we share our experience of providing ART in two conflict affected settings in Africa and the Middle-east. The aim of this paper is summarize different operational approaches that have been successfully implemented in different contexts experiencing periods of acute instability, the Central African Republic (CAR) and Yemen.

## Case Presentation

### Central African Republic

#### Context

CAR has endured decades of instability, trapped in a cycle of constant and interminable conflict since the late 1990s. In some parts of the country as much as half the population are affected by conflict, with many displaced from their homes. The negative health effects of these conflicts are considerable, in addition to impacting schooling for children, agricultural production, access to functioning markets and the degradation of essential infrastructure including roads.

HIV prevalence in CAR is among the highest in Central and Western Africa, estimated at 4.9–10% amongst adults according to different UN sources [14, 15]. Before a surge in violence in 2013, around 15,000 people living with HIV (PLWH) were on ART. The biggest concern for the HIV programme was interruption of ART due to stock ruptures and loss of HIV patients on treatment due to insecurity and displacement. Both events may have affected thousands of PLWH and could have led to increased mortality and the development of drug resistance. Possibly reflecting these risks, rates of treatment failure in CAR are high and estimated at 30% in adults and 50% in children [16, 17]. More than a fifth (3000) of patients experiencing treatment failure had developed drug resistance. The ability of the health system to handle ART failure is insufficient, with few qualified health workers, no second-line ART available, extremely limited access to virological monitoring, and frequent and long-term nationwide shortages of medical supplies [18]. Adding to these concerns, the breakdown of law and order led to widespread violations of human rights including sexual and gender based violence and rape [19].

**Fig. 1:**
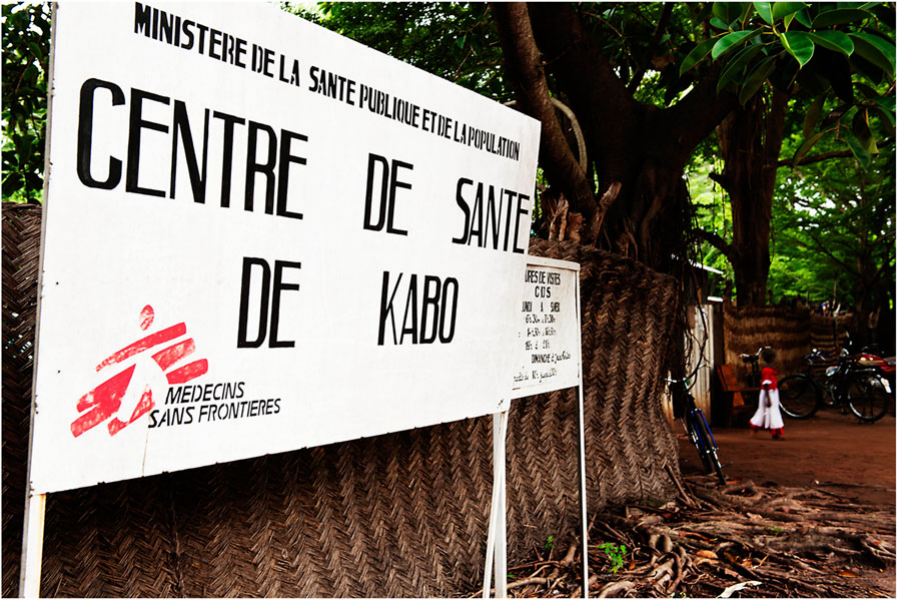
MSF Project Kabo, CAR

MSF Spain has been present in CAR since 1996 in the north and central part of the country – Kabo, Batangafo and Ndele towns – 3 rural areas chronically affected by conflict (Fig. 1). In 2008 HIV care was integrated into the existing health services.

#### Program design and implementation

HIV services began by addressing patients presenting at the inpatient department (IPD), malnourished children, patients with tuberculosis (TB) or any sign suggesting HIV infection, and prevention of mother-to-child transmission (PMTCT) at antenatal clinics (ANC). Later in 2010, a voluntary counselling and testing service (VCT) and health promotion activities were added.

HIV protocols and ART eligibility criteria were in line with the National AIDS Program (NAP) until 2012 when, with the agreement from Ministry of Health (MOH), MSF introduced tenofovir (TDF) based fixed-dose combinations as first line therapy to replace existing stavudine (d4T) based regimens following 2010 WHO recommendations [20]. Option B+ for PMTCT was implemented in 2014. In November 2016 the NAP adopted the WHO recommendations to start ART to all individuals as soon as possible following an HIV diagnosis; however implementation of such recommendations is still very slow.

Implementation of these activities was mostly organised by a HIV experienced international doctor providing training to national and international staff, implementing protocols, setting up data collection systems, and monitoring activities. HIV testing and counselling (HTC) was performed by trained community healthcare workers (CHW) using rapid test kits, while clinical consultations were run by nurses supported by a doctor in complicated cases. HIV and TB consultations were integrated into the OPD. Tracing of defaulters was done by CHWs and included tracing of patients under TB treatment, although these activities where not always done due to limitations on access linked with insecurity.

Clinical monitoring was done every 1–3 months depending on adherence and security conditions. No regular access to CD4 or viral load was available. Point of care devices to measure creatinine and haemoglobin were available to support ARV regimen choices. There was no NAP electronic database and MOH ART registers were used to report a list of indicators entered into the Health Information System (HIS) used by MSF Spain. Tracing of patients lost to care was not always possible due to security concerns, so the outcomes of many patients lost to care are not known.

Overall, 1820 patients were diagnosed with HIV and enrolled into care between 2008 and 2016. 1440 (79%) patients initiated ART, including 90 (8%) children. Median age was 31.7 years old, 1231 (67%) patients were classified as WHO stage 3 or 4 at admission and 77% of the patients started on ART were women. 538 (37%) of the enrolled patients underwent CD4 cell count testing; 333 (61.8%) had CD4 cell count < 200 cell/mm^3^, 217 (12%) CD4 cell count 200–500 cells/mm^3^ and 50 (2.7%) patients had a CD4 cell count > 500 cells/mm^3^. By September 2013, when the context became increasingly unstable, 683 (60%) patients remained under ART and active follow-up.

#### Contingency planning

A contingency plan to ensure continuation of ART in case of insecurity was implemented in 2010 covering different security scenarios, from low levels of insecurity with short-term break in services up to full evacuation of most of the staff and distribution of “run-away” packs containing 2 extra months of ARVs plus a 1 week tail protection with AZT/3TC in cases of high insecurity [11]. Patients were informed about what to do in case of violence during routine counselling sessions and national staff were trained on how to prepare in case of evacuation.

**Fig. 2:**
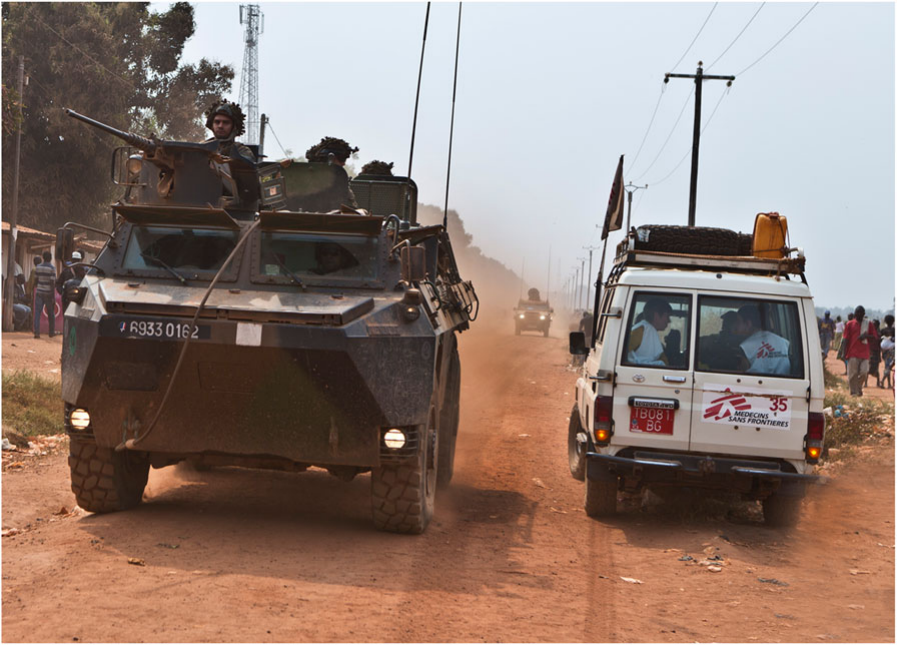
MSF response during acute conflict in CAR 2013

In September 2013 the security situation deteriorated, with several armed attacks displacing the population in the MSF supported region with many people crossing the border to Chad. During the initial phase of the plan, patients came to the MSF-supported health clinics to receive extra ARVs (2 months’ supply) and were provided information about what to do in case treatment interruption occurred given the unpredictable situation. There were several patients living in more remote areas who could not reach the clinic and MSF staff could not move, leaving a number of patients’ without access to drugs (Fig 2).

In January 2014 the international MSF team had to be evacuated and all HIV services where put on hold except for PMTCT that continued with the national staff that remained in the Hospital. HIV activities where reinitiated in July 2014 when situation became more stable and the MSF team could return.

The contingency plan was rapidly implemented to ensure continuation of treatment in case of population movement or MSF evacuation. Run away bags were distributed to 594 (87%) patients during the peak of violence between September and December 2013. By February 2014, 313 (52%) of those patients had returned for consultation. By December 2016, since the beginning of the programme 782 (54%) patients started on ART were still under active follow up, 354 (25%) patients had been lost to follow up and 182 (13%) had died. Only 26 patients (0.1%) had received a second line regimen. These outcomes, while not optimal, are in line with outcomes reported over similar time periods in stable settings: a recent report from 57 cohorts from 22 countries found that 52.1% of patients were retained on ART, 41.8% were lost to follow-up and 6.0% had died 5 years after ART initiation [21].

During 2016 viral load monitoring was implemented by sending dried blood spot samples to an external laboratory in South Africa; during this time 390 samples were sent and 212 (54%) results were available. 139 (66%) patients were virologicaly suppressed and 71 (33%) had a viral load higher than 1000 copies/ml. Those with a high VL were provided enhanced adherence support and switched to second line if needed.

### Yemen

#### Context

Yemen is frequently affected by intra-state conflicts. The HIV epidemic is described as a mature, low-level epidemic with prevalence estimated at 0.2% in the general population; however Yemen has a concentrated HIV epidemic among men who have sex with men (MSM) with an estimated prevalence of 5.9%. It is estimated that at the end of 2013 there were 35,000 people living with HIV in Yemen [22].

**Fig. 3:**
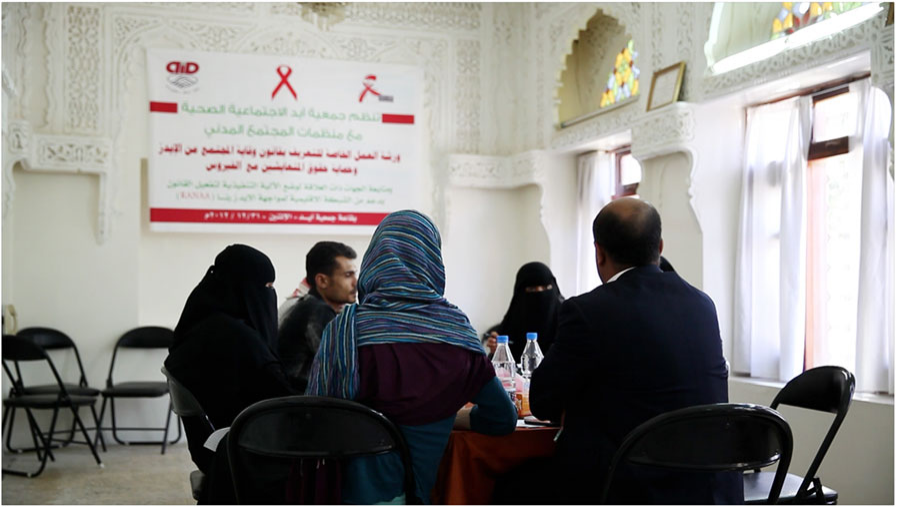
MSF HIV Project Sanaa, Yemen

MSF has been present in Yemen since 2007 in the Awhar region covering Abyan and Shabwa Governorates with medical programs responding to violence. In February 2009 an assessment of access to HIV services found high levels of stigma and discrimination; patients were being rejected from the hospitals because of their positive status, surgeries had been denied and HIV-positive pregnant women had to attend private clinics to deliver. In response, MSF decided to start HIV services supporting the National AIDS Programme in the capital of Sana’a with the main objective of increasing access to HIV care and reducing stigma and discrimination (Fig. 3).

#### Program design and implementation

The main HIV activities were to provide support to the National AIDS Programme in Sana’s city at the ART clinic in Al-Ghomouri Hospital. MSF support included training of national staff, covering ARV stock ruptures and a major focus on health promotion and advocacy efforts towards reduction of stigma and discrimination aimed at the community, patients and hospital staff.

HIV testing was available at the clinic and patients could self-present or be referred. Community outreach activities including health promotion were carried out to increase HIV awareness and testing. HIV protocols and ART eligibility criteria were in line with the National AIDS Program (NAP) and in 2010 a TDF based fixed-dose combination as first line was implemented to replace existing stavudine-based regimens following WHO recommendations [20].

Since the war in 2015, a policy of “Treat all” was implemented by the NAP to facilitate treatment initiation of patient’s diagnosed HIV positive and avoid loss to follow up of patients prior to ART.

HIV activities were initially implemented by international staff that performed mentoring activities with national doctors at the government HIV clinic in Sana’a. Clinical trainings were provided to medical and non-medical staff of the hospital as a way to increase awareness about HIV and decrease stigma. Since 2011 due to the unstable situation, the MSF HIV program has been entirely run by national staff.

Patients were reviewed monthly for the first year after ART initiation, then every 3 months depending on adherence, per national guidelines. CD4 was done at baseline and every 6 months on treatment; targeted viral load was used to detect treatment failure but was not accessible for routine monitoring. Point of care devices to measure creatinine and haemoglobin were used to support ARV regimen choice. In 2010 MOH implemented Tier.net as an M&E tool for the entire country, starting with Sana’a as pilot project [23].

Since the beginning of the project in 2010, 1655 patients were diagnosed HIV positive, 1470 (89%) were started on ART, 126 (9%) were lost to follow up, 288 (20%) died and 1056 (72%) are still under care as of December 2016. Thirty seven patients (4%) are receiving a second line regimen. When the crisis broke out in 2011, there were 363 patients under ART.

#### Contingency planning

In February 2011, government loyalists and opposition tribesmen clashed during protests in Yemen, especially in the capital Sana’a, against the regime of President Ali Abdullah Saleh. The situation subsequently devolved into open fighting between military forces loyal to the government, defecting military forces, and tribal militia in the capital Sana’a in May 2011.

Unlike in CAR, there was no contingency plan defined at the beginning of the program and the team was forced to develop a plan to ensure ART continuation. All HIV services were interrupted, including HIV testing and ART initiation for new patients.

All patients received a “health card” describing the ARV regimen they were receiving, what to do in case of inability to reach the health facility, and an emergency phone number to call in case they ran out of drugs. The nurse in charge of the pharmacy removed the patient’s registers and ARVs from the clinic to his house and started calling all patients to deliver run-away bags and managed the emergency phone line. Patients called the phone number if they had any questions prior to the next drug refill and arranged with the MSF team drug distribution in different areas of the city. An extra ART site was allocated out of the area most affected by the clashes where patients could go to pick up drugs. This system lasted for 7 months and patients could call the MSF nurse if they were running out of drugs or needed psychosocial support. A contingency plan was developed building on these lessons to prepare for future episodes of instability and shared with the National AIDS Programme.

Between April and November 2011, 363 (100%) patients received run-away bags with an extra 2 months of drugs and by February 2012, 354 (98%) patients had returned for their follow up consultation. No patient reported having had to interrupt their treatment.

After activities resumed in 2014 the war started again in April 2015, at which point the contingency plan was again enacted, with the support from peer patients and associations of PLWH, whose work helped ensure the continuation of treatment for patients. This time patients were given 3–4 months of drugs to try and minimise the risk of treatment disruptions.

## Conclusions

Our experience adds to the growing evidence that provision of HIV services in conflict-affected settings is feasible with simplified models and strategies and should be included in the comprehensive medical response in conflict-affected populations. This includes contingency plans to respond in case of emergencies implemented from the beginning of the intervention and linked with proper counselling and patient information. Simplified protocols are consistent with a public health approach to ART delivery [24] and have been successfully applied in other African countries where basic laboratory commodities and human resources are limited, without compromising quality.

This experience demonstrates the value of implementing different strategies according to the challenges: in places with high HIV prevalence and low resources available, a step-wise approach initially targeting the sickest patients (e.g. those admitted to IPDs, malnutrition centres and TB wards) could be the preferred option until capacity is built to extend care to all HIV patients. In places where a lower number of HIV patients are expected, strategies targeting all HIV infected populations in all medical departments could be feasible.

In our program both strategies were tailored to the setting. CAR with a higher HIV prevalence started targeting the sicker patients while in Yemen a full package of HIV activities was implemented from the beginning of the intervention. In both settings the inclusion of a focal person for the implementation of HIV activities (e.g. training of national staff, setting up of basic activities and supervision of the HIV program) was an added value and it allowed a more rapid start of activities compared to other projects where there have not been any extra person to start HIV care.

Outcomes described in this case study are similar to other non-conflict affected African countries [25], although rates of loss to follow up were higher in CAR particularly after the 2013 peak of violence possibly related to the high mobility of the population. Retention in care and viral suppression in CAR was similar to other reports in African countries without security issues. It is important to note that few patients are receiving second line regimens in our programs mainly due to the limited availability of virological monitoring to detect treatment failure.

In Yemen the outcomes of the contingency plan were better than those observed in CAR despite not having a plan beforehand. This difference could be related to the fact that Sana’a is an urban area where patients can still look for a safer place and it has more reliable access to telephone and communication networks, whereas the rural areas of CAR without access to phone networks and not many options to seek safer places, makes this setting more challenging to ensure access of and to patients.

These results have in part been achieved by helping to empower and educate patients since the beginning of the intervention and encouraging them to be responsible for their own treatment. Still today, very few actors and donors are willing to support HIV services in countries chronically affected by violence and HIV packages are rarely included in the initial response to emergencies, despite a consensus statement made in 2006 by WHO, UNHCR, UNAIDS, MSF and UNICEF that ARV delivery should be included as part of comprehensive HIV services in emergency settings [26].

Rates of loss to follow up are much higher in CAR despite improved counselling approaches and adequate defaulter tracing systems; this might be due to the frequent episodes of violence in the area and the continuous internal displacement.

Preferably, contingency plans should be in place from the beginning of the programme, prior to the onset of the emergency. Nevertheless, our experience in Yemen shows that even in the absence of a prior contingency plan, simple actions can be taken rapidly to ensure continuity of care. Pharmacy management should also be included with the contingency plan to ensure the availability of enough drugs for the times of acute instability when drugs for 4–6 months are given to patients. This could jeopardize further supply if not planned beforehand.

The recommendation to treat all HIV positive patients regardless of clinical or immunological status provides an opportunity to improve access to ART in conflict settings where very often there is no access to CD4 or viral load monitoring and ART coverage lies far behind other countries in the Sub-Saharan Africa region. Differentiated models of care including simplification of protocols, task shifting, patient and community involvement [27], and contingency planning are additional elements that support ART delivery in such settings.

There is a need to ensure access to HIV services, including ART in conflict settings which have some of the lowest rates of treatment coverage world-wide. Places like South Sudan, or most of the Western and Central African countries (WCA) are among the regions with ART coverage below 50% [28]; improved access to HIV services are urgently needed in these settings if we want to reach the UNAIDS 90–90-90 targets. Funding and support to implement these activities is a major concern when other “competing priorities” are prioritized and main donors maintain a focus of funding for specific fields of interest. Donor support could be crucial to close the gaps when talking about ART services in acute emergencies [29]. Our case studies demonstrate the importance and feasibility of providing HIV care in conflict affected populations in both low and high prevalence HIV settings. Simplified protocols and strategies tailored to the context should be thought about when providing access to health care in these settings.

## Abbreviations

ANC: Antenatal care
ART: Antiretroviral treatment
ARV: Antiretroviral
CAR: Central African Republic
CHW: Community health worker
D4T: Stavudine
HIS: Health information system
HIV: Human immunodeficiency virus
HTC: HIV testing and counselling
IPD: Inpatients department
M&E: Monitoring and evaluation
MOH: Ministry of Health
MSF: Médecins sans Frontières
MSM: Men who have sex with men
NAP: National AIDS Program
OPD: Outpatient department
PLWHA: People living with HIV AIDS
PMTCT: Prevention mother-to-child transmission
TB: Tuberculosis
TDF: Tenofovir
UN: United Nations
VCT: Voluntary counselling and testing
VL: Viral load
WCA: Western and Central African region
WHO: World health organization
